# Image Compressive Sensing via Hybrid Nonlocal Sparsity Regularization

**DOI:** 10.3390/s20195666

**Published:** 2020-10-03

**Authors:** Lizhao Li, Song Xiao, Yimin Zhao

**Affiliations:** State Key Lab of Integrated Services Networks, Xidian University, Xi’an 710071, China; lz_li@stu.xidian.edu.cn (L.L.); zhaoyimin@stu.xidian.edu.cn (Y.Z.)

**Keywords:** compressive sensing, nonlocal self-similarity, sparse representation

## Abstract

This paper focuses on image compressive sensing (CS). As the intrinsic properties of natural images, nonlocal self-similarity and sparse representation have been widely used in various image processing tasks. Most existing image CS methods apply either self-adaptive dictionary (e.g., principle component analysis (PCA) dictionary and singular value decomposition (SVD) dictionary) or fixed dictionary (e.g., discrete cosine transform (DCT), discrete wavelet transform (DWT), and Curvelet) as the sparse basis, while single dictionary could not fully explore the sparsity of images. In this paper, a Hybrid NonLocal Sparsity Regularization (HNLSR) is developed and applied to image compressive sensing. The proposed HNLSR measures nonlocal sparsity in 2D and 3D transform domain simultaneously, and both self-adaptive singular value decomposition (SVD) dictionary and fixed 3D transform are utilized. We use an efficient alternating minimization method to solve the optimization problem. Experimental results demonstrate that the proposed method outperforms existing methods in both objective evaluation and visual quality.

## 1. Introduction

**Figure 1:**
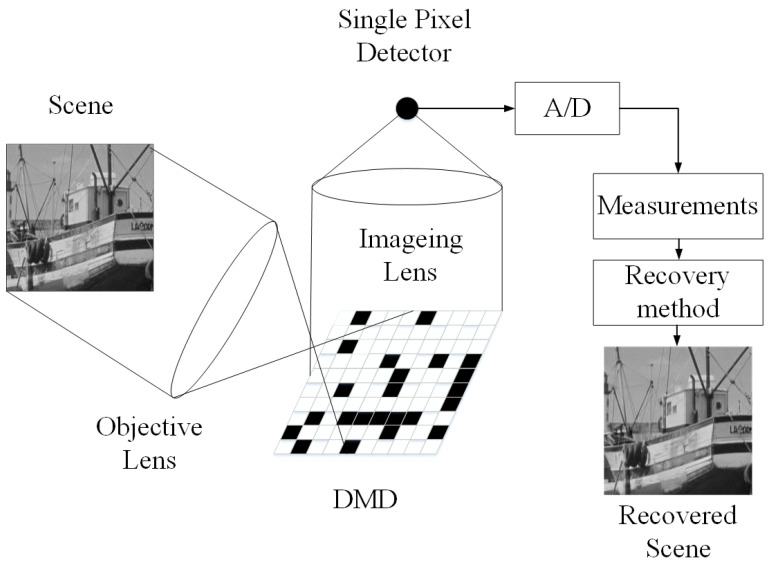
Architecture of the single-pixel camera [3].

As a joint framework of sampling and compression, compressive sensing (CS) [1,2] shows that if a signal is sparse in some domains, it can be perfectly reconstructed from fewer samples than Nyquist rate. This characteristic demonstrates its two great potentials in signal acquisition and processing. First, as the number of samples is greatly reduced, this make it possible for some devices with limited sensor size to obtain high definition information using low definition sensors. Figure 1 shows the architecture of the single-pixel camera [3]. With a sensor with only one pixel, this system can get a complete image. Second, the CS framework transfers the computational burden to the decoding side. For some energy limited applications, such as wireless sensor network, this advantage can greatly extend the life cycle of the nodes. As the encoding side is simplified, the performance of the system depends largely on the performance of the decoding side, namely, the “Recovery method” part in Figure 1. This paper focus on the recovery method of image CS. Due to the advantages mentioned above, CS have been applied in many fields, such as digital imaging [3], background subtraction [4], medical imaging [5], and remote sensing [6].

In the framework of compressive sensing, a one-dimensional sparse signal can be reconstructed by solving a L0-norm minimization problem. Since L0-norm minimization is non-convex and NP-hard, L0-norm is often replaced by L1-norm. It has been proved that these two norm are equivalent in most cases [2] and many CS recovery methods are proposed, such as iterative thresholding algorithm [7], orthogonal matching pursuit [8], and split Bregman algorithm [9].

For image compressive sensing, the key issue is how to exploit the intrinsic prior information of images. As the model of prior knowledge has a significant impact on the performance of image compressive sensing algorithms, many kinds of regularizations have been developed. Conventional regularization terms, such as Mumford–Shah (MS) model [10] and total variation (TV) [7,11,12,13], are established under the assumption that images are locally smooth. For example, Li et al. [13] proposed a TV-based CS algorithm and developed an efficient augmented lagrangian method to solve it. Candes et al. [11] enhanced the sparsity of TV norm via a weighted strategy. However, these regularizations only consider local smoothness of images and cannot restore details and textures well. TV norm also favors piecewise constant solution, resulting in oversmoothing. To overcome this problem and improve performance, many compressive sensing methods utilized the prior information of transform coefficients [14,15,16]. Kim et al. [15] modeled the statistical characteristics between transform coefficients with a Gaussian Scale Mixture (GSM) and achieved better reconstruction performance.

In the past few years, sparse representation has begun to emerge and demonstrated good performance in various image processing tasks [17,18,19,20,21]. The purpose of sparse representation is to represent a signal with as few atoms as possible in a learned over-complete dictionary. Compared with fixed dictionary, the learned dictionary can better express sparsity of images. However, dictionaries are generally learned from external clean images, and it may suffer from high computational complexity.

Recently, inspired by nonlocal means (NLM) [22], many algorithms based on nonlocal self-similarity have been proposed [23,24,25,26,27,28,29]. Dabov et al. proposed a Block-Matching and 3D filtering (BM3D) algorithm for image denoising [23]. In BM3D, similar patches in a degraded image are grouped into 3D arrays and collaborative filtering is performed in 3D transform domain. Egiazarain et al. extended BM3D to compressive sensing and proposed BM3D-CS. Zhang et al. [26] proposed a structural group sparsity representation (SGSR) model to enforce image sparsity in an adaptive SVD domain. Dong et al. [28] proposed a nonlocal low-rank regularization (NLR) to exploit the self-similarity, and applied it to the reconstruction of photographic and MRI images. In [29], Zha et al. incorporated a non-convex penalty function to group sparse representation and obtained state-of-the-art reconstruction performance. Gao et al. [30] proposed to use Z-score standardization to improve the sparse representation ability of patch groups. Keshavarzian et al. [31] proposed to utilize the principle component analysis (PCA) to learn a dictionary for each group and introduced non-convex LLp-norm regularization to better promote the sparsity of the patch group coefficients. In [32], internal self-adaptive dictionary and external learned dictionary were used to encode a patch group alternately and achieved better performance than single dictionary.

Another idea is to exploit both local sparsity and nonlocal self-similarity [33,34,35,36,37]. For example, Zhang et al. [33] combined local anisotropic total variation with nonlocal 3D sparsity, and named it Collaborative Sparsity Measure (CoSM). Different from the work in [33], Eslahi et al. [37] used curvelet transform to enforce local patterns. In [34], Dong et al. utilized local patch-based sparsity and nonlocal self-similarity constrain to balance the trade-off between adaptation and robustness. Zhou, et al. [38] proposed a data-adaptive kernel regressor to extract local structure and used nonlocal means filter to enforce nonlocal information.

With the development of deep learning, many convolutional neural network (CNN) based image compressive sensing algorithms have been proposed. For example, Kulkarni et al. [39] proposed a non-iterative and parallelizable CNN architecture to get an initial recovery and fed it into an off-the-shelf denoiser to obtain the final image. Zhang et al. [40] cast the Iterative Shrinkage- Thresholding Algorithm (ISTA) into CNN framework and developed an effective strategy to solve it. In [41], low-rank tensor factor analysis was utilized to capture nonlocal correlation and a deep convolutional architecture was adopted to accelerate the matrix inversion in CS. DR${}^{2}$-Net [42] utilized a linear mapping to reconstruct a preliminary image and used residual learning to further promote the reconstruction quality. Yang et al. [43] unrolled the Alternating Direction Method Multipliers (ADMM) to be a deep architecture and proposed ADMM-CSNet. Zhang et al. [44] proposed a optimization-inspired explicable deep network OPINE-Net and all the parameters were learned end-to-end using back-propagation.

**Figure 2:**
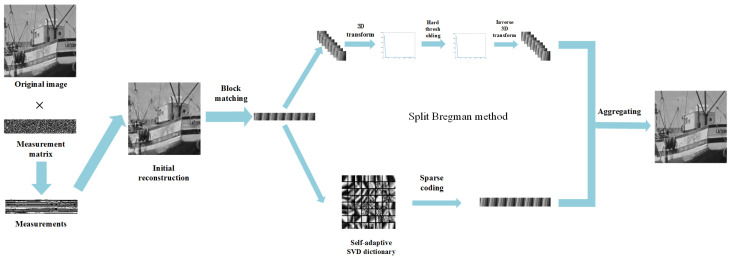
Flowchart of the proposed HNLSR-CS.

In this paper, we propose a Hybrid NonLocal Sparsity Regularization (HNLSR) for image compressive sensing. First, different from the methods mentioned above, two nonlocal self-similarity constrains are applied to exploit the intrinsic sparsity of images simultaneously. Then, fixed dictionaries are universal, and learned dictionaries are more robust to the image itself. To take advantages of them, both fixed 3D transform and 2D self-adaptive dictionary are utilized. Finally, for the non-convex model of HNLSR, we use the split Bregman to divide it into several subproblems, making it easier and more efficient to be solved. The flowchart is illustrated in Figure 2. Experimental results show that compared with both model-based algorithms and deep learning-based algorithms, the proposed HNLSR-CS demonstrates the superiority of its performance.

The remainder of this paper is organized as follows. Section 2 introduces the related works. In Section 3, we present the proposed method. The experiment and analysis are elaborated in Section 4. Section 5 concludes the paper.

## 2. Related Work

### 2.1. Compressive Sensing

For a n-dimension signal $x\in R^{n}$, its CS measurements can be expressed as

(1) y=Φx

where $y\in R^{m}$ and $\Phi \in R^{m\times n}\left(m\ll n\right)$. Φ is the measurement matrix which meets the restricted isometry property (RIP) [1]. If x is sparse in a transform domain $\Psi \in R^{n\times n}$, namely, x=Ψα, the reconstruction of x can be formulated as

(2) $$\hat{\alpha }=\underset{\alpha }{\arg\min}{∥\alpha ∥}_{0}\;s.t.\;y=\Phi \Psi \alpha$$

where ${∥\alpha ∥}_{0}$ is the L0-norm that counts the nonzero elements in α.

The unconstrained Lagrangian form of Equation (2) is

(3) $$\hat{\alpha }=\underset{\alpha }{\arg\min}\frac{1}{2}{∥y-\Phi \Psi \alpha ∥}_{2}^{2}+\lambda {∥\alpha ∥}_{0}$$

where λ is the regularization parameter. After getting the solution of Equation (4), x can be restored by $\hat{x}=\Psi \Psi \hat{\alpha }$.

For image compressive sensing, the optimization problem can be written as

(4) $$\hat{x}=\underset{x}{\arg\min}\frac{1}{2}{∥y-\Phi x∥}_{2}^{2}+R\left(x\right)$$

where x stands for an image, Φ is the measurement matrix and R(x) is the regularization item which exploits the intrinsic prior information of images.

### 2.2. Sparse Representation and Group-Based Sparsity

For an image $x\in R^{N}$, it can be divided into many overlapped patches. Suppose a patch $x_{i}$ of size $\sqrt{n}\times \sqrt{n}$ at location *i*, i=1,2,…,N, sparse representation means that this patch can be represented over a redundant dictionary $D_{i}$

(5) $$\hat{\alpha_{i}}=\underset{\alpha_{i}}{\arg\min}{∥\alpha_{i}∥}_{0}\;s.t.\;x_{i}=D_{i}\alpha_{i}$$

Nonlocal self-similarity means that a patch has many similar patches in other positions [18,22,23]. We search its (m−1) best matched patches and form them into a data matrix $x_{G_{i}}\in R^{n\times m}$, where each column of $x_{G_{i}}$ denotes a similar patch, so we have

(6) $$x_{G_{i}}=R_{G_{i}}\left(x\right)$$

where subscript $G_{i}$ is the number of the group, $R_{G_{i}}$ is an operator that extract all the similar patches and $x_{G_{i}}$ is a patch group. Given a proper dictionary $D_{G_{i}}$, this group can be expressed as

(7) $$\hat{\alpha_{G_{i}}}=\underset{\alpha_{G_{i}}}{\arg\min}{∥\alpha_{G_{i}}∥}_{0}\;s.t.\;x_{G_{i}}=D_{G_{i}}\alpha_{G_{i}}$$

where $\hat{\alpha_{G_{i}}}$ is the sparse coefficient. After getting $\hat{\alpha_{G_{i}}}$, the whole image can be reconstructed via [45]

(8) $$x\approx {\left(\sum_{i}^{N}R_{G_{i}}^{T}1_{\left(n\times m\right)}\right)}^{-1}\left(\sum_{i}^{N}R_{G_{i}}^{T}D_{G_{i}}\hat{\alpha_{G_{i}}}\right)$$

where $1_{\left(n\times m\right)}$ is a matrix of size n×m with all the elements being 1. Equation (8) means that we can restore the image by putting patches back to their original locations and averaging them on a pixel-by-pixel basis.

### 2.3. Nonlocal Self-Similarity in 3D Transform Domain

Dabov et al. proposed the well-known BM3D [23] for image denoising and the self-similarity in 3D transform domain has attracted great attention since then [24,33,37]. For a patch $x_{i}$ of size $\sqrt{n}\times \sqrt{n}$, after searching its (m−1) similar patches, they are stacked into a 3D array *Z* of size $\sqrt{n}\times \sqrt{n}\times m$. Next, a 3D transform is performed to get the transform coefficients

(9) $$T_{3D}\left(Z\right)=\Theta$$

where $T_{3D}\left(\cdot \right)$ is a transform operator and Θ are coefficients. Since these coefficients are considered sparse, they are shrunken by some filters (e.g., soft-thresholding or hard-thresholding). Then, the sparse coefficients are inverted to generate the estimated group. These estimates are returned to their original positions. Nonlocal 3D sparsity can explore high degree sparsity of images, and can well preserve details and differences between patches.

### 2.4. Split Bregman Iteration

The split Bregman iteration (SBI) [9] was proposed to solve various optimization problem. Considering a constrained problem:

(10) $$\min_{x,z}H\left(x\right)+G\left(z\right)\;s.t.x=Kz$$

where $H:R^{N}\to R$, $G:R^{M}\to R$ and $K^{N\times M}\in R^{N\times M}$. H(·) and G(·) are convex functions. This optimization problem can be efficiently solved by Algorithm 1. According to the SBI framework, as x and z have some relationship, the optimization problem can be split into two subproblem (namely, step 3 and step 4). The rationale behind is that in step 3 and step 4, only one variable is solved at a time, making it much easier than the original problem.
**Algorithm 1** Split Bregman Iteration (SBI).  1:**Set**μ, k=0, $x^{\left(0\right)}=0$, $z^{\left(0\right)}=0$, $b^{\left(0\right)}=0$.  2:**repeat**  3: $x^{\left(k+1\right)}=\underset{x}{\arg\min}H\left(x\right)+\frac{\mu }{2}{∥x-Kz^{\left(k\right)}-d^{\left(k\right)}∥}_{2}^{2}$;  4: $z^{\left(k+1\right)}=\underset{z}{\arg\min}G\left(z\right)+\frac{\mu }{2}{∥x^{\left(k+1\right)}-Kz-d^{\left(k\right)}∥}_{2}^{2}$;  5: $d^{\left(k+1\right)}=d^{\left(k\right)}-\left(x^{\left(k+1\right)}-Kz^{\left(k+1\right)}\right)$;  6: k=k+1.  7:**until** Stop criterion is satisfied.

## 3. Proposed Method

### 3.1. Hybrid Non-Local Sparsity Regularization (Hnlsr)

Integrating two kinds of different nonlocal regularizations, we propose a Hybrid Non-Local Sparsity Regularization (HNLSR), and it can be expressed as

(11) $$R_{HNLSR}\left(x\right)=\lambda {∥\alpha ∥}_{0}+\tau {∥T_{3D}\left(Z(x)\right)∥}_{1}=\lambda {∥\alpha ∥}_{0}+\tau {∥\Theta ∥}_{1}$$

where α are the coefficients under certain 2D sparse dictionary and λ and τ are regularization parameters. Z(x) is the 3D form of *x*. The proposed regularization has two advantages:

1. It constrains sparsity in both 2D and 3D domains, which means that it can better explore the intrinsic nonlocal similarity of images.
2. We use a self-adaptive dictionary as the 2D sparse basis and a fixed 3D transform to measure sparsity in high-dimensional space. Two kinds of different dictionaries can improve the robustness of the regularization.

Next, we will apply the proposed HNLSR to image compressive sensing and show how to solve the optimization problem.

### 3.2. Image Cs Via Hnlsr

Incorporating Equation (11) into Equation (4), the proposed optimization problem for image CS is expressed as

(12) $$\begin{array}{c} \hat{x}=\min_{x}\frac{1}{2}{∥y-\Phi x∥}_{2}^{2}+\lambda ∥\alpha ∥{}_{0}+\tau {∥\Theta ∥}_{1} \end{array}$$

where λ and τ are regularization parameters. We use the SBI framework to solve this optimization problem. Define $H\left(x\right)=\frac{1}{2}{∥y-\Phi x∥}_{2}^{2}$ and $G\left(z\right)=\lambda {∥\alpha ∥}_{0}+\tau {∥\Theta ∥}_{1}$, so the corresponding *K* are sparse dictionaries. Invoking Line 3 in Algorithm 1, we obtain

(13) $$\begin{array}{cc} x^{\left(k+1\right)} & =\underset{x}{\arg\min}\frac{1}{2}{∥y-\Phi x∥}_{2}^{2}+\frac{\mu }{2}{∥x-Dz^{\left(k\right)}-d^{\left(k\right)}∥}_{2}^{2}\\ \; & =\underset{x}{\arg\min}\frac{1}{2}{∥y-\Phi x∥}_{2}^{2}+\frac{\mu }{2}{∥\left[x,x\right]-\left[D_{2D},D_{3D}\right]\left[\begin{array}{c} \alpha^{\left(k\right)}\\ \Theta^{\left(k\right)} \end{array}\right]-\left[\begin{array}{c} b^{\left(k\right)}\\ c^{\left(k\right)} \end{array}\right]∥}_{2}^{2} \end{array}$$

where $d^{\left(k\right)}=\left[\begin{array}{c} b^{\left(k\right)}\\ c^{\left(k\right)} \end{array}\right]$. Splitting the second term in Equation (13), we have

(14) $$x^{\left(k+1\right)}=\underset{x}{\arg\min}\frac{1}{2}{∥y-\Phi x∥}_{2}^{2}+\frac{\mu_{1}}{2}{∥x-D_{2D}\alpha^{\left(k\right)}-b^{\left(k\right)}∥}_{2}^{2}+\frac{\mu_{2}}{2}{∥x-D_{3D}\Theta^{\left(k\right)}-c^{\left(k\right)}∥}_{2}^{2}$$

Then we apply Line 4 and Equation (12) is transformed into

(15) $$\begin{array}{cc} z^{\left(k+1\right)} & =\underset{z}{\arg\min}G\left(z\right)+\frac{\mu }{2}{∥x^{\left(k+1\right)}-Dz-d^{\left(k\right)}∥}_{2}^{2}\\ \; & =\underset{\alpha ,\Theta }{\arg\min}\lambda {∥\alpha ∥}_{0}+\tau {∥\Theta ∥}_{1}+\frac{\mu }{2}{∥\left[x^{\left(k+1\right)},x^{\left(k+1\right)}\right]-\left[D_{2D},D_{3D}\right]\left[\begin{array}{c} \alpha \\ \Theta \end{array}\right]-\left[\begin{array}{c} b^{\left(k\right)}\\ c^{\left(k\right)} \end{array}\right]∥}_{2}^{2} \end{array}$$

Finally, b and c can be calculated by

(16) $$b^{\left(k+1\right)}=b^{\left(k\right)}-\left(x^{\left(k+1\right)}-D{}_{2D}\alpha^{\left(k+1\right)}\right)$$

(17) $$c^{\left(k+1\right)}=c^{\left(k\right)}-\left(x^{\left(k+1\right)}-D{}_{3D}\Theta^{\left(k+1\right)}\right)$$

Therefore, the minimization problem of Equation (12) is divided into several subproblems and the solution to each subproblem will be discussed below.

#### 3.2.1. x-Subproblem

Given α, Θ, b and c, Equation (14) is a convex quadratic function optimization problem and we can use gradient descent method to solve this problem efficiently

(18) $$x^{\left(k+1\right)}=x^{\left(k\right)}-\eta^{\left(k\right)}g^{\left(k\right)}$$

where $g^{\left(k\right)}$ is the gradient direction of Equation (14)

(19) $$g^{\left(k\right)}=\Phi^{T}\Phi x^{\left(k\right)}-\Phi^{T}y+\mu_{1}\left(x^{\left(k\right)}-D_{2D}\alpha^{\left(k\right)}-b^{\left(k\right)}\right)+\mu_{2}\left(x^{\left(k\right)}-D_{3D}\Theta^{\left(k\right)}-c^{\left(k\right)}\right)$$

and $\eta^{\left(k\right)}$ is the optimal step-size and calculated via

(20) $$\eta =\frac{g^{T}g}{g^{T}\Phi^{T}\Phi g+\left(\mu_{1}+\mu_{2}\right)g^{T}g}$$

The superscript *k* of g is omitted for conciseness.

#### 3.2.2. z-Subproblem

Given x, b and c, z-subproblem Equation (15) can be divided into two formulas

(21) $$\alpha^{\left(k+1\right)}=\underset{\alpha }{\arg\min}\lambda {∥\alpha ∥}_{0}+\frac{\mu_{1}}{2}{∥x^{\left(k+1\right)}-D_{2D}\alpha -b^{\left(k\right)}∥}_{2}^{2}$$

(22) $$\Theta^{\left(k+1\right)}=\underset{\Theta }{\arg\min}\tau {∥\Theta ∥}_{1}+\frac{\mu_{2}}{2}{∥x^{\left(k+1\right)}-D_{3D}\Theta -c^{\left(k\right)}∥}_{2}^{2}$$

Let us define ${\bar{x}}^{\left(k+1\right)}=x^{\left(k+1\right)}-b^{\left(k\right)}$, where ${\bar{x}}^{\left(k+1\right)}$ can be seen as the noisy observation of $x^{\left(k+1\right)}$. Therefore, Equation (21) can be rewritten as

(23) $$\alpha^{\left(k+1\right)}=\underset{\alpha }{\arg\min}\lambda {∥\alpha ∥}_{0}+\frac{\mu_{1}}{2}{∥{\bar{x}}^{\left(k+1\right)}-D_{2D}\alpha ∥}_{2}^{2}$$

As patch group is the basic unit of sparse coding, this problem can be split into divided into several subproblems. Moreover, for each subproblem, the coefficients of each group are the variables to be solved. Therefore, Equation (23) can be solved by

(24) $$\min_{\alpha }\sum_{m=1}^{n}\left(\frac{1}{2}{∥{\bar{x}}_{G_{m}}^{\left(k+1\right)}-D_{2D_{m}}\alpha_{G_{m}}∥}_{F}^{2}+\theta {∥\alpha_{G_{m}}∥}_{0}\right)$$

where $\theta =\frac{\lambda }{\mu_{1}}$ and ${\bar{x}}_{G_{m}}^{\left(k+1\right)}$ is image patch group. $D_{2D_{m}}$ and $\alpha_{G_{M}}$ are corresponding dictionary and sparse coefficients. For every group, we adopt the singular value decomposition (SVD) to generate the 2D dictionary. Applying the SVD to a group ${\bar{x}}_{G_{m}}$, we have

(25) $${\bar{x}}_{G_{m}}=U_{G_{m}}\Sigma_{G_{m}}V_{G_{m}}^{T}$$

where $\Sigma_{G_{m}}$ is a diagonal matrix formed by the eigenvalues. Moreover, the dictionary is defined as

(26) $$D_{G_{m}}=U_{G_{m}}V_{G_{m}}^{T}$$

Therefore, for every optimization problem in Equation (24), it has a close-form solution

(27) $$\alpha_{G_{m}}^{i}=hard\left(\Sigma_{G_{m}}^{i},\sqrt{2\theta }\right)=\Sigma_{G_{m}}^{i}⊙1\left(abs\left(\Sigma_{G_{m}}^{i}\right)-\sqrt{2\theta }\right)$$

where hard(·) is hard thresholding function and ⊙ stands for the element-wise product operator.

Similar to the α-subproblem, we define ${\hat{x}}^{\left(k+1\right)}=x^{\left(k+1\right)}-c^{\left(k\right)}$ and consider the fact that the probability of every overlapped image patch appearing is equal, we can solve Equation (22) by

(28) $$\min_{\Theta }\sum_{m=1}^{n}\left(\frac{1}{2}{∥{\hat{x}}_{G_{m}}^{\left(k+1\right)}-D_{3D}\Theta_{G{}_{m}}∥}_{F}^{2}+\frac{\tau }{\mu_{2}}{∥\Theta_{G_{m}}∥}_{1}\right)$$

where ${\hat{x}}_{G_{m}}^{\left(k+1\right)}$ is a 3D patch array. This problem can be seen as a filtering problem in transform domain. Invoking the Bayesian framework [21], the maximum a posterior (MAP) estimation of $\Theta_{G_{m}}$ with ${\hat{x}}_{G_{m}}^{\left(k+1\right)}$ is

(29) $$\begin{array}{cc} \Theta_{G_{m}} & =\underset{\Theta_{G_{m}}}{\arg\max}\log P(\Theta_{G_{m}}\left|{\hat{x}}_{G_{m}}^{\left(k+1\right)})\right.\\ \; & =\underset{\Theta_{G_{m}}}{\arg\max}\left\{\log P\left({\hat{x}}_{G_{m}}^{\left(k+1\right)}\left|\Theta_{G_{m}}\right.\right)+\log P\left(\Theta_{G_{m}}\right)\right\} \end{array}$$

Assuming that ${\hat{x}}_{G_{m}}^{\left(k+1\right)}$ is disturbed by Gaussian noise with standard deviation $\sigma_{n}$ and $\Theta_{G_{m}}$ follows i.i.d Laplacian distribution

(30) $$P\left(\Theta_{G_{m}}\right)=\prod_{i}\left\{\frac{1}{\sqrt{2}\sigma^{i}}\exp\left(-\frac{\left|\Theta_{G_{m}}^{i}\right|}{\sigma_{i}}\right)\right\}$$

where $\sigma_{i}$ is the standard deviations of $\Theta_{G_{m}}^{i}$. Substituting Equation (30) into Equation (29), we can obtain

(31) $$\underset{\Theta_{G_{m}}}{\arg\min}\frac{1}{2}{∥{\hat{x}}_{G_{m}}^{\left(k+1\right)}-D_{3D}\Theta_{G_{m}}∥}_{F}^{2}+2\sqrt{2}\sigma_{n}^{2}\times \sum_{i=1}^{l}\frac{1}{\sigma_{i}}\left|\Theta_{G_{m}}^{i}\right|$$

From the above analysis, we can know that $\frac{\tau }{\mu_{2}}=\frac{2\sqrt{2}\sigma_{n}^{2}}{\sigma_{i}}$ and Equation (31) can be solved by soft thresholding function

(32) $$\Theta_{G_{m}}=\mathrm{sgn}\left(D_{3D}^{T}{\hat{x}}_{G_{m}}^{\left(k+1\right)}\right)\cdot \max\left(\left|D_{3D}^{T}{\hat{x}}_{G_{m}}^{\left(k+1\right)}\right|-\frac{2\sqrt{2}\sigma_{n}^{2}}{\sigma_{i}},0\right)$$

The proposed method for image compressive sensing is summarized in Algorithm 2.
**Algorithm 2** Image compressive sensing via HNLSR.  1:**Input:** measurement y, measurement matrix Φ**Initialization**: (1) Set *k*, λ, τ, b, c, *m*, $\sigma_{n}$;(2) Estimate an initial image $x_{init}$;  2:Compute x via Eq.(19);  3:**for** Each patch **do**  4: (1) Block-matching and form patch group;  5: (2) Generate dictionary for every patch group via Eq.(25);  6: (3) Compute α via Eq.(27);  7:**end for**  8:**for** Each patch **do**  9: (1) Search similar patches and arrange as 3D arrays;10: (2) Compute Θ via Eq.(32);11:**end for**12:Update b via Eq.(16);13:Update c via Eq.(17);14:k=k+1.15:**Output:** Reconstructed image $x^{*}$

## 4. Experimental Results

### 4.1. Implementation Details

**Figure 3:**
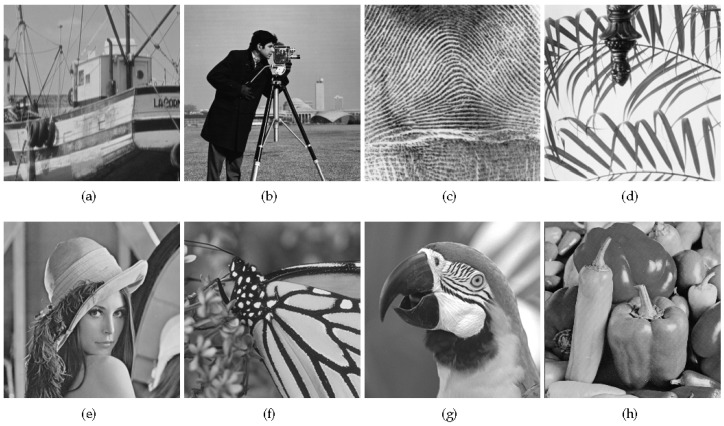
Eight test images. (**a**) Boats. (**b**) Cameraman. (**c**) Fingerprint. (**d**) Leaves. (**e**) Lena. (**f**) Monarch. (**g**) Parrots. (**h**) Peppers.

This section presents the performance of the proposed HNLSR methods. In our experiment, eight commonly used images are used to test the reconstruction performance of the algorithms (shown in Figure 3). The size of them is 256×256. In the measurement phase, a image is divided into blocks of size 32×32 and Gaussian matrix is applied to generate measurements for each block. In the reconstruction phase, the size of overlapping patches is 8×8. Step size, i.e., the distance between two image patches in the horizontal or vertical direction, is set as 4. For every image patch, we search its 59 similar patches in a 20×20 window. $\mu_{1}$ and $\mu_{2}$ are set to (0.0025, 0.0025), (0.0025, 0.00025), and (0.0025, 0.0001) when the sampling rates are 0.1, 0.2, and 0.3, respectively. The 3D dictionary is composed of 2D DCT and 1D Haar wavelet. Maximum iteration number is 120. We use peak signal-to-noise ratio (PSNR)and feature similarity (FSIM) [46] as the performance evaluation indices. All experiments are performed in Matlab R2017a on computer with Intel Core i5-6500 CPU of 3.2 Ghz, 8 GB memory, and Windows 10 operating system.

### 4.2. Comparison with State-of-the-Art Methods

We compare our method with six representative methods: MH-BCS [47], RCoS [33], ALSB [27], GSR [45], JASR [37], and GSR-NCR [29]. MH-BCS uses residual in the measurement domain and multihypothesis predictions to improve reconstruction quality; RCoS utilizes nonlocal 3D sparsity and local 2D sparsity (namely, total variation (TV)) to explore the intrinsic of images; ALSB is a patch-based sparse representation method; JASR employs discrete curvelet transform (DCuT) to constrain local sparsity and combines it with nonlocal 3D sparsity; GSR is an extended version of SGSR [26]. Both GSR and GSR-NCR are group-based method, and their difference is GSR uses L0-norm to constrain the sparse coefficients, while GSR-NCR uses non-convex Lp-norm. GSR and GSR-NCR are known as the stat-of-the-art methods. The PSNR and FSIM results are shown in Table 1 and Table 2 respectively, and the best result for each sampling rate is marked in bold.

We can see that compared with MH-BCS, methods based on non-local self-similarity have obvious advantages in performance. As a patch-based algorithm, ALSB is inferior to other methods in most cases. JASR performs better than RCoS since DCuT is better than TV in depicting local characteristics. Compared with methods using fixed dictionaries (namely RCoS and JASR), methods using self-adaptive dictionaries have better performance in general. The proposed method combines fixed dictionary with self-adaptive dictionary and get the best performance in most cases.

**Figure 4:**
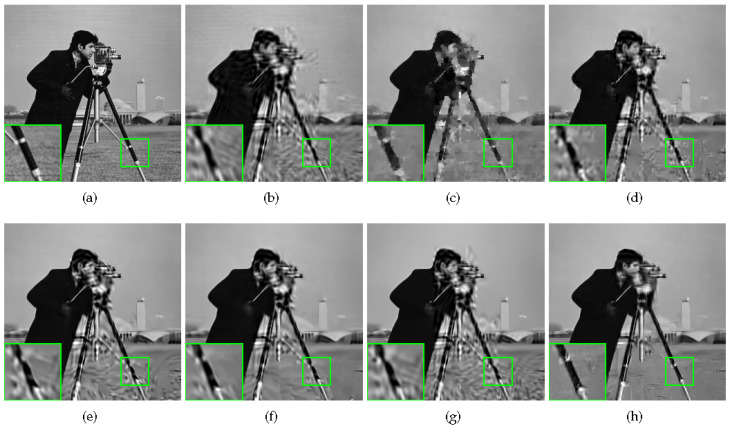
Reconstruction of Cameraman with sampling rate = 0.1. (**a**) Original image; (**b**) MH (PSNR = 22.13 dB, FSIM = 0.7692); (**c**) RCoS (PSNR = 22.97 dB, FSIM = 0.7942); (**d**) ALSB (PSNR = 22.97 dB, FSIM = 0.8021); (**e**) GSR (PSNR = 22.89 dB, FSIM = 0.8154); (**f**) JASR (PSNR = 23.54 dB, FSIM = 0.8139); (**g**) GSR-NCR(PSNR = 22.50 dB, FSIM = 0.8012); (**h**) Proposed HNLSR (PSNR = **24.67** dB, FSIM = **0.8408**)).

**Figure 5:**
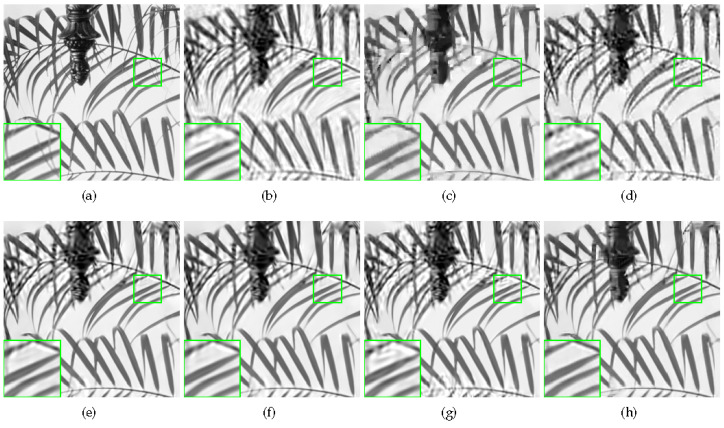
Reconstruction of Leaves with sampling rate = 0.1. (**a**) Original image; (**b**) MH (PSNR = 20.89 dB, FSIM = 0.7634); (**c**) RCoS (PSNR = 22.38 dB, FSIM = 0.8632); (**d**) ALSB (PSNR = 21.32 dB, FSIM = 0.7916); (**e**) GSR (PSNR = 23.22 dB, FSIM = 0.8755); (**f**) JASR (PSNR = 23.62 dB, FSIM = 0.8799); (**g**) GSR-NCR(PSNR = 22.26 dB, FSIM = 0.8408); (**h**) Proposed HNLSR (PSNR = **24.54** dB, FSIM = **0.8984**)).

**Figure 6:**
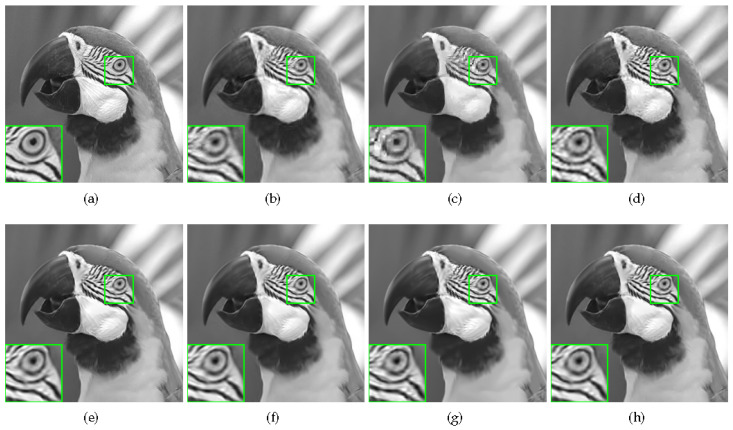
Reconstruction of Parrots with sampling rate = 0.2. (**a**) Original image; (**b**) MH (PSNR = 29.23 dB, FSIM = 0.9405); (**c**) RCoS (PSNR = 28.61 dB, FSIM = 0.9311); (**d**) ALSB (PSNR = 29.73 dB, FSIM = 0.9460); (**e**) GSR (PSNR = 31.17 dB, FSIM = 0.9524); (**f**) JASR (PSNR = 31.09 dB, FSIM = 0.9478); (**g**) GSR-NCR(PSNR = 30.18 dB, FSIM = 0.9435); (**h**) Proposed HNLSR (PSNR = **31.41** dB, FSIM = **0.9526**)).

**Figure 7:**
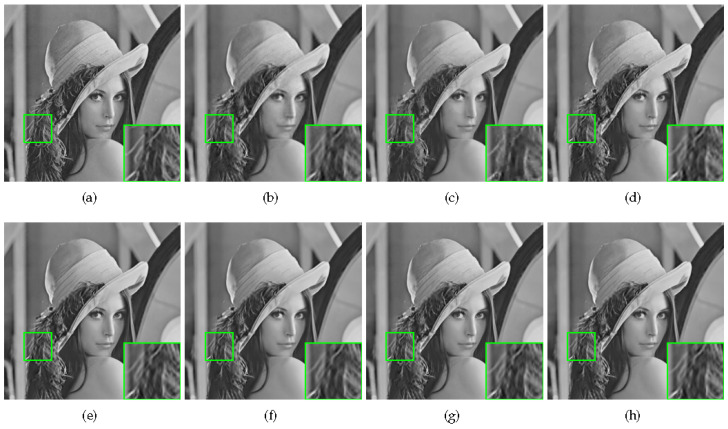
Reconstruction of Lena with sampling rate = 0.3. (**a**) Original image; (**b**) MH (PSNR = 31.99 dB, FSIM = 0.9538); (**c**) RCoS (PSNR = 32.41 dB, FSIM = 0.9555); (**d**) ALSB (PSNR = 33.30 dB, FSIM = 0.9650); (**e**) GSR (PSNR = 34.17 dB, FSIM = **0.9716**); (**f**) JASR (PSNR = 34.05 dB, FSIM = 0.9677); (**g**) GSR-NCR(PSNR = 33.94 dB, FSIM = 0.9715); (**h**) Proposed HNLSR (PSNR = **34.27** dB, FSIM = **0.9716**)).

Some visual comparisons are illustrated in Figure 4, Figure 5, Figure 6 and Figure 7. In Figure 4, it is obvious that MH-BCS generates the worst result. ALSB, GSR, and GSR-NCR suffer from some artifacts in the water surface area. RCoS and JASR have better results, but the edge of the tripod is a little blurry. In Figure 5, other methods produce some undesirable traces in the blank area, and the proposed method is not only pure in the blank area, but also has relatively sharp leaf edges. MH-BCS, RCoS, and ALSB produce some unexpected noise in the white area around the eyes in Figure 6, and the pattern around the eyes of the proposed method is the clearest. It is evident that in terms of visual quality, the proposed method outperforms other methods.

We also compare the HNLSR-CS with three representative deep learning methods: ReconNet [39], ISTA-Net${}^{+}$ [40], and DR${}^{2}$-Net [42]. We use pretrained models for testing and the PSNR and FSIM results are reported in Table 3 and Table 4. The best results are highlighted in bold. The proposed method obtains the best result in most cases.

**Figure 8:**
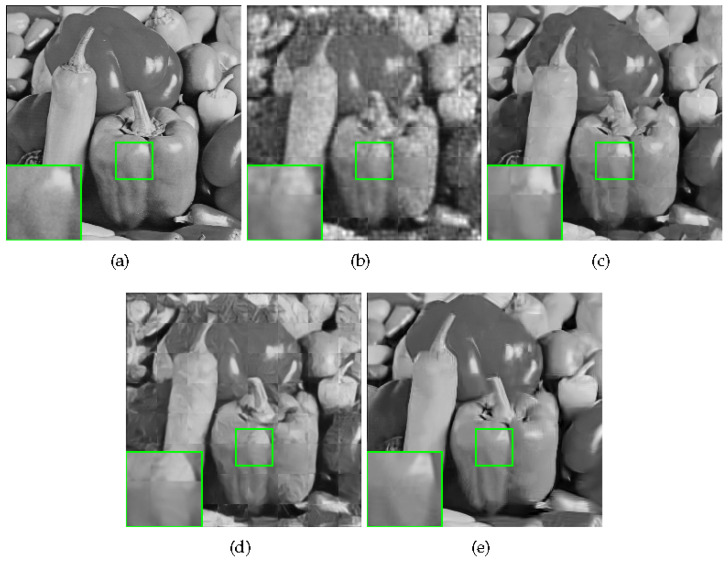
Reconstruction of Monarch with sampling rate = 0.1. (**a**) Original image; (**b**) ReconNet (PSNR = 22.14 dB, FSIM = 0.7840); (**c**) ISTA-Net${}^{+}$ (PSNR = 27.23 dB, FSIM = 0.8862); (**d**) DR${}^{2}$-Net (PSNR = 23.73 dB, FSIM = 0.8282); (**e**) Proposed HNLSR (PSNR = **27.91** dB, FSIM = **0.8962**).

**Figure 9:**
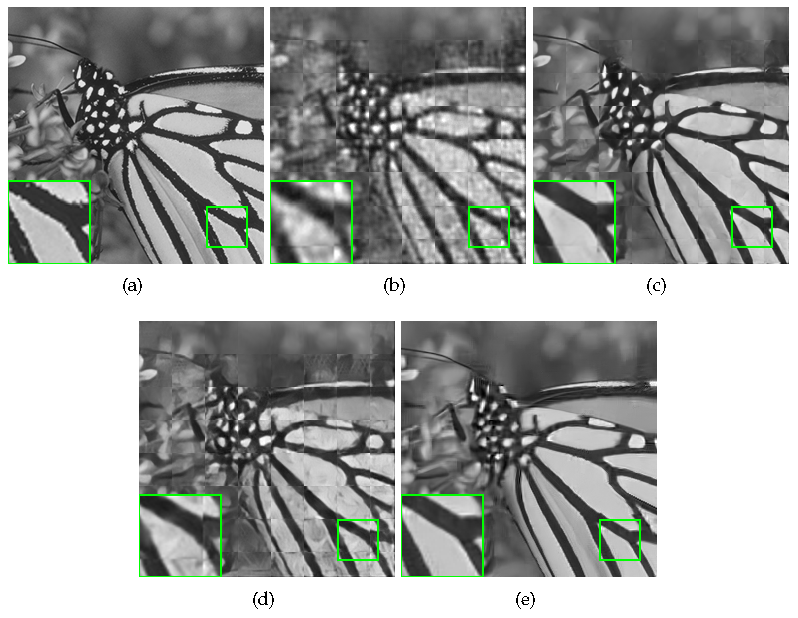
Reconstruction of Monarch with sampling rate = 0.1. (**a**) Original image; (**b**) ReconNet (PSNR = 21.11 dB, FSIM = 0.7406); (**c**) ISTA-Net${}^{+}$ (PSNR = **26.58 dB**, FSIM = 0.8816); (**d**) DR${}^{2}$-Net (PSNR = 23.10 dB, FSIM = 0.8184); (**e**) Proposed HNLSR (PSNR = 26.26 dB, FSIM = **0.8907**).

Some visual comparisons are shown in Figure 8 and Figure 9. In Figure 8, ReconNet, ISTA-Net${}^{+}$, and DR${}^{2}$-Net all suffer from block effects, and the proposed method has the best details. In Figure 9, ReconNet, and DR${}^{2}$-Net still have some block artifacts; ISTA-Net${}^{+}$ has the best PSNR, but it produces some undesirable artifacts, resulting in worse FSIM than ours. These results also prove the superiority of the proposed method.

### 4.3. Effect of Parameters of Similar Patches

**Figure 10:**
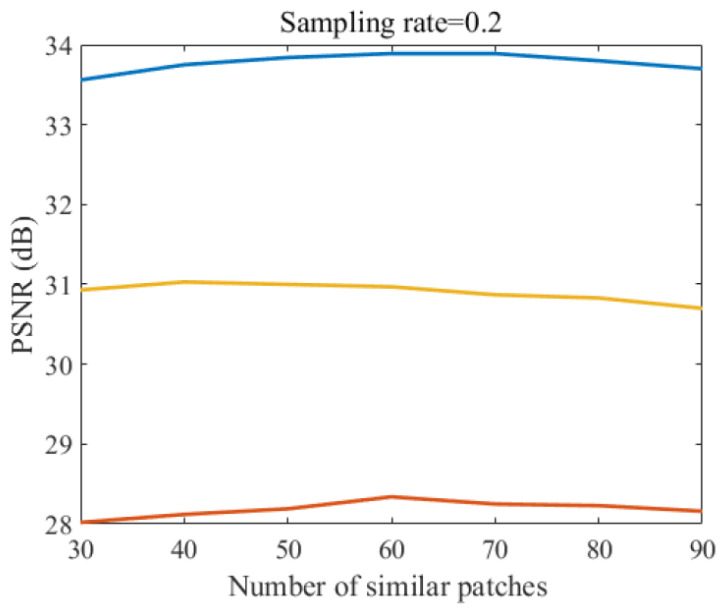
Performance comparison with different number of patches for three test images in case of sampling rate = 0.2.

In this section, we discuss how the parameters of similar patches affects the performance of the method. With other variables fixed, we change the number of similar patches at intervals of 10 between 30 and 90. The comparisons are shown in Figure 10. We can see from the figure that all three curves are relatively stable, which means that the performance is not sensitive to the number of image patches. Considering the performance and complexity of the method, we set the number of similar patches to 60.

### 4.4. Convergence

**Figure 11:**
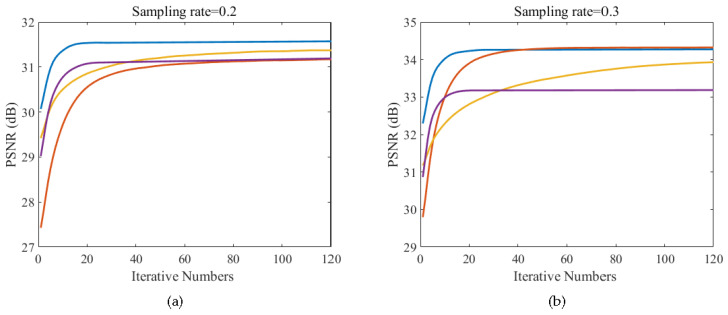
Evolutions of PSNR versus iteration number for four test images. (**a**) Sampling rate = 0.2; (**b**) Sampling rate = 0.3.

As Equation (12) is non-convex, it is difficult to give a theoretical proof of the convergence of the proposed method, so we only show its stability through empirical evidence. Figure 11 shows the curve of PSNR versus iteration number of four images at the sampling rate of 0.2 and 0.3, respectively. We can see from the figure that with the iteration number increases, PSNR changes drastically at the beginning, and then gradually become stable. This illustrates the good convergence performance of the proposed method.

## 5. Conclusions and Future Work

This paper proposes a Hybrid Nonlocal Sparsity Regularization (HNLSR) method for image compressive sensing. Different from existing methods, the proposed HNLSR does not consider the local sparsity of images, but uses two dictionaries to explore the nonlocal self-similarity. The 2D dictionary is self-generated and the 3D dictionary is a fixed dictionary, which can combine the advantages of adaptability and versatility from different dictionaries. An effective framework based on SBI is present to solve the optimization problem. The convergence and stability of the proposed method have also been proven. Experimental results show that compared with methods which are based on local and nonlocal regularizations or single nonlocal regularization, the proposed method performs better than most existing image compressive sensing methods in both quality assessment and visual quality.

As multiple dictionaries can improve the performance, we are considering some research directions. For example, learning different dictionaries for different areas of the images (e.g., smooth area and textured area). Another direction is to learn multi-scale dictionaries and select them adaptively according to the parameters. Our future work include extending the proposed method to other image processing tasks (e.g., denoising, deblocking, and deblurring) and high-dimensional data (e.g., videos and multispectral images). For high-dimensional or multi-frame data, how to collect similar patches (intra- or inter-frame) is also a problem to be solved.

## Figures and Tables

**Table 1:** PSNR(dB) comparison of six representative methods and the proposed method.

Rate	Methods	Boats	C.man	F.print	Leaves	Lena	Monarch	Parrots	Peppers	Average
0.1	MH-BCS	26.11	22.13	20.08	20.89	26.13	23.19	25.34	25.00	23.61
	RCoS	27.85	22.97	16.30	22.38	27.53	25.56	25.60	27.41	24.45
	ALSB	28.12	22.97	20.68	21.32	27.04	24.34	26.03	26.67	24.65
	GSR	28.30	22.89	20.27	23.22	27.56	25.29	26.37	26.91	25.10
	JASR	28.59	23.54	21.04	23.62	27.90	25.83	26.76	27.60	25.61
	GSR-NCR	27.96	22.50	20.50	22.26	27.02	24.67	26.03	26.37	24.66
	Proposed HNLSR	**28.77**	**24.67**	**21.12**	**24.54**	**28.04**	**26.26**	**27.22**	**27.91**	**26.07**
0.2	MH-BCS	29.91	25.88	23.17	25.14	29.81	27.10	29.23	28.45	27.34
	RCoS	31.42	25.68	19.64	27.22	30.36	29.60	28.61	30.87	27.93
	ALSB	33.27	26.65	23.64	26.97	30.73	28.30	29.73	29.87	28.65
	GSR	33.69	27.17	23.85	30.54	31.36	30.78	31.17	30.83	29.92
	JASR	32.70	27.75	23.98	30.24	31.19	30.60	31.09	31.06	29.83
	GSR-NCR	33.30	26.30	23.67	29.03	30.87	29.46	30.18	20.46	29.16
	Proposed HNLSR	**33.89**	**28.34**	**24.03**	**30.97**	**31.57**	**31.17**	**31.41**	**31.19**	**30.32**
0.3	MH-BCS	32.25	28.08	24.73	27.63	31.99	27.10	31.01	30.30	29.14
	RCoS	34.32	27.98	22.74	30.92	32.41	32.53	30.53	32.65	30.51
	ALSB	36.59	29.01	25.81	31.01	33.30	31.41	31.98	32.13	31.41
	GSR	36.91	29.62	26.20	34.46	34.17	34.25	33.81	33.02	32.81
	JASR	36.08	29.93	26.21	33.70	34.05	33.63	33.10	33.09	32.47
	GSR-NCR	**37.27**	29.37	**26.35**	**34.95**	33.94	**34.68**	33.07	32.86	32.81
	Proposed HNLSR	36.94	**30.01**	26.27	34.54	**34.27**	34.27	**33.93**	**33.18**	**32.93**

**Table 2:** FSIMcomparison of six representative methods and the proposed method.

Rate	Methods	Boats	C.man	F.print	Leaves	Lena	Monarch	Parrots	Peppers	Average
0.1	MH-BCS	0.8489	0.7692	0.8512	0.7634	0.8913	0.7912	0.8981	0.8489	0.8328
	RCoS	0.8765	0.7942	0.6027	0.8632	0.8863	0.8757	0.8919	0.8794	0.8337
	ALSB	0.8934	0.8021	0.8682	0.7916	0.8965	0.8251	0.9105	0.8735	0.8576
	GSR	0.9027	0.8154	0.8691	0.8755	**0.9147**	0.8673	**0.9229**	0.8859	0.8817
	JASR	0.9035	0.8139	**0.8722**	0.8799	0.9107	0.8822	0.9176	0.8918	0.8840
	GSR-NCR	0.898	0.8012	0.8688	0.8408	0.9106	0.8318	0.9190	0.8733	0.8679
	Proposed HNLSR	**0.9042**	**0.8408**	0.8622	**0.8984**	0.9092	**0.8907**	0.9204	**0.8962**	**0.8903**
0.2	MH-BCS	0.9159	0.8552	0.9103	0.8577	0.9348	0.8751	0.9405	0.9036	0.8991
	RCoS	0.9348	0.8645	0.7923	0.9307	0.9331	0.9314	0.9311	0.9281	0.9058
	ALSB	0.9522	0.8759	0.9208	0.9069	0.9440	0.8907	0.9460	0.9228	0.9199
	GSR	0.9581	0.8946	0.9254	0.9559	0.9537	0.9411	0.9524	0.9332	0.9393
	JASR	0.9458	0.8961	0.9256	0.9516	0.9434	0.9409	0.9478	0.9342	0.9342
	GSR-NCR	0.9526	0.8797	0.9225	0.9430	0.9470	0.9216	0.9435	0.9268	0.9296
	Proposed HNLSR	**0.9589**	**0.9096**	**0.9271**	**0.9586**	**0.9545**	**0.9454**	**0.9526**	**0.9364**	**0.9429**
0.3	MH-BCS	0.9439	0.8938	0.9331	0.8961	0.9538	0.899	0.9563	0.9269	0.9254
	RCoS	0.9615	0.9089	0.8937	0.9579	0.9555	0.9555	0.9501	0.9472	0.9413
	ALSB	0.9748	0.9190	0.9471	0.9508	0.9650	0.9303	0.9620	0.9455	0.9493
	GSR	0.9770	0.9325	0.9520	0.9765	**0.9716**	0.9636	0.9668	0.9513	0.9614
	JASR	0.9723	0.9311	0.9510	0.9719	0.9677	0.9610	0.9623	0.9505	0.9585
	GSR-NCR	**0.9783**	0.9305	**0.9534**	**0.9799**	0.9715	**0.9668**	0.9660	0.9501	0.9621
	Proposed HNLSR	0.9772	**0.9366**	0.9523	0.9769	**0.9716**	0.9639	**0.9670**	**0.9525**	**0.9623**

**Table 3:** PSNR (dB) comparison of deep learning methods and the proposed method.

Rate	Methods	Boats	C.man	F.print	Leaves	Lena	Monarch	Parrots	Peppers	Average
0.04	ReconNet	21.36	19.26	14.67	15.40	21.28	18.19	20.27	19.56	18.75
	ISTA-Net${}^{+}$	22.23	20.45	14.99	16.38	22.64	19.54	21.97	21.47	19.96
	DR${}^{2}$-Net	22.11	19.84	**15.04**	16.29	22.13	18.93	21.16	20.31	19.48
	Proposed HNLSR	**23.22**	**21.09**	14.90	**18.08**	**24.52**	**20.49**	**23.46**	**23.49**	**21.16**
0.1	ReconNet	24.15	21.28	15.84	18.35	23.83	21.11	22.63	22.14	21.17
	ISTA-Net${}^{+}$	27.44	23.66	17.47	23.44	27.65	**26.58**	26.58	27.23	25.01
	DR${}^{2}$-Net	25.58	22.46	17.21	20.26	25.39	23.10	23.94	23.73	22.71
	Proposed HNLSR	**28.77**	**24.67**	**21.12**	**24.54**	**28.04**	26.26	**27.22**	**27.91**	**26.07**
0.25	ReconNet	27.30	23.15	19.10	21.91	26.54	24.32	25.59	24.77	24.09
	ISTA-Net${}^{+}$	33.71	29.19	23.47	31.96	32.70	**33.41**	31.99	**32.70**	31.14
	DR${}^{2}$-Net	30.09	25.62	21.63	25.65	29.42	27.95	28.73	28.49	27.20
	Proposed HNLSR	**35.44**	**29.34**	**25.14**	**33.25**	**33.08**	33.36	**32.79**	32.41	**31.85**

**Table 4:** FSIM comparison of deep learning methods and the proposed method.

Rate	Methods	Boats	C.man	F.print	Leaves	Lena	Monarch	Parrots	Peppers	Average
0.04	ReconNet	0.7310	0.6954	0.5873	0.6122	0.7641	0.6833	0.7835	0.7327	0.6987
	ISTA-Net${}^{+}$	0.7616	0.7300	0.5781	0.6876	0.8003	0.7403	0.8235	0.7806	0.7378
	DR${}^{2}$-Net	0.7574	0.7134	**0.6013**	0.6770	0.7869	0.7217	0.7991	0.7587	0.7269
	Proposed HNLSR	**0.7805**	**0.7501**	0.5717	**0.7617**	**0.8389**	**0.7734**	**0.8732**	**0.8136**	**0.7704**
0.1	ReconNet	0.7910	0.7440	0.6714	0.6835	0.8137	0.7406	0.8285	0.7840	0.7571
	ISTA-Net${}^{+}$	0.8756	0.8289	0.7007	0.8760	0.8967	0.8816	0.9062	0.8862	0.8565
	DR${}^{2}$-Net	0.8415	0.7896	0.7305	0.7948	0.8488	0.8184	0.8605	0.8282	0.8140
	Proposed HNLSR	**0.9042**	**0.8408**	**0.8622**	**0.8984**	**0.9092**	**0.8907**	**0.9204**	**0.8962**	**0.8903**
0.25	ReconNet	0.8730	0.8030	0.8166	0.7765	0.8765	0.8152	0.8801	0.8460	0.8359
	ISTA-Net${}^{+}$	0.9575	0.9205	0.9111	0.9623	0.9583	**0.9607**	0.9560	**0.9491**	0.9469
	DR${}^{2}$-Net	0.9198	0.8575	0.8793	0.8902	0.9200	0.8989	0.9204	0.9034	0.8987
	Proposed HNLSR	**0.9699**	**0.9244**	**0.9427**	**0.9715**	**0.9646**	0.9605	**0.9602**	0.9469	**0.9551**
